# Construction of a novel signature based on immune-related lncRNA to identify high and low risk pancreatic adenocarcinoma patients

**DOI:** 10.1186/s12876-023-02916-y

**Published:** 2023-09-14

**Authors:** Na Li, Jionghuang Chen, Weihua Yu, Xiaoling Huang

**Affiliations:** 1 Nursing Department, Sir Run Run Shaw Hospital, Zhejiang University School of Medicine, Hangzhou, China; 2 Department of General Surgery, Sir Run Run Shaw Hospital, Zhejiang University School of Medicine, Hangzhou, China

**Keywords:** PAAD, irlncRNAs, Prognostic risk model, Tumor immune microenvironment

## Abstract

**Background:**

Pancreatic adenocarcinoma is one of the most lethal tumors in the world with a poor prognosis. Thus, an accurate prediction model, which identify patients within high risk of pancreatic adenocarcinoma is needed to adjust the treatment and elevate the prognosis of these patients.

**Methods:**

We obtained RNAseq data of The Cancer Genome Atlas (TCGA) pancreatic adenocarcinoma (PAAD) from UCSC Xena database, identified immune-related lncRNAs (irlncRNAs) by correlation analysis, and identified differential expressed irlncRNAs (DEirlncRNAs) between pancreatic adenocarcinoma tissues from TCGA and normal pancreatic tissues from TCGA and Genotype-Tissue Expression (GTEx). Further univariate and lasso regression analysis were performed to construct prognostic signature model. Then, we calculated the areas under curve and identified the best cut-off value to identify high- and low-risk patients with pancreatic adenocarcinoma. The clinical characteristics, immune cell infiltration, immunosuppressive microenvironment, and chemoresistance were compared between high- and low-risk patients with pancreatic adenocarcinoma.

**Results:**

We identified 20 DEirlncRNA pairs and grouped the patients by the best cut-off value. We proved that our prognostic signature model possesses a remarkable efficiency to predict prognosis of PAAD patients. The AUC for ROC curve was 0.905 for 1-year prediction, 0.942 for 2-year prediction, and 0.966 for 3-year prediction. Patients in high-risk group have poor survival rate and worse clinical characteristics. We also proved that patients in high-risk groups were in immunosuppressive status and may be resistant to immunotherapy. Anti-cancer drug evaluation was performed based on in-silico predated tool, such as paclitaxel, sorafenib, and erlotinib, may be suitable for PAAD patients in high-risk group.

**Conclusions:**

Overall, our study constructed a novel prognostic risk model based on pairing irlncRNAs, exhibited a promising prediction value in patients with pancreatic adenocarcinoma. Our prognostic risk model may help distinguish PAAD patients suitable for medical treatments.

## Introduction

Pancreatic cancer is a malignant tumor with low five-year survival rate and high malignancy. At the time of diagnosis, most patients are already in advanced stages. Under the background of the COVID-19 epidemic, doctors and nurses are under great pressure when treating patients with pancreatic cancer, and the family members of patients also face multiple pressures when participating in the treatment decision-making [1, 2]. Although much improvements have been made in the treatment of PAAD, such as neoadjuvant therapy, surgical resection, radiotherapy, chemotherapy, targeted molecular therapy and immune checkpoint inhibitors (ICIs), only about 9% of patients can live for five years after diagnosis [3, 4]. Due to the atypical initial symptoms of pancreatic adenocarcinoma, patients are generally diagnosed with advanced stage and accompanied by metastasis [5]. Hence, for a certain patient, an individualized comprehensive treatment should weigh the advantages and disadvantages of all treatment options not only prolong survival but also improves quality of life [6]. Therefore, an effective prediction model is needed for the accurate assessment of patient’s prognosis [7]. In this way, appropriate treatments may be taken to balance the survival benefits and the quality of life in PAAD patients.

The poor prognosis of PAAD is mainly due to the tolerance to chemotherapeutic drugs. In recent years, immune checkpoint inhibitors have been widely used in the treatment of solid tumors [8]. However, the use of ICIs in pancreatic adenocarcinoma is rarely successful [9]. Thus, it is very important to identify patients which could benefit from ICIs treatment.

Long-noncoding RNAs (lncRNAs) are one kind of non-coding RNAs with transcripts > 200 nucleotides. LncRNAs are extensive, accounting for about 80% of the human transcriptome [10]. A body of work has indicated that prognosis models based on lncRNAs could effectively predict the prognosis of patients [11, 12]. For example, 18 autophagy-related lncRNAs were identified to construct a prognostic signature in breast cancer [13]. Other immune-related 6 lncRNAs were used to establish a prognostic signature in glioma [14].

In pancreatic adenocarcinoma, some researches have established lncRNAs-based signatures to predicted prognosis of patients. A 3-lncRNAs signature was established in pancreatic adenocarcinoma, with its area under the ROC curve (AUC) only 0.742 for an overall survival (OS) of 3 years [15]. In addition, the lncRNA expression values changes in different gene sets, different data forms and different patients, the scores of prognosis model are unstable. Thus, we utilized a novel modeling algorithm, paring, and iteration to construct an immune-related lncRNAs (irlncRNA) signature, to construct a more accurate and stable prognosis model [8].

## Methods

### Collection of data and identification of DEirlncrna

The RNAseq normalized data (FPKM)and clinical data of pancreatic adenocarcinoma of TCGA and Genotype-Tissue Expression (GTEx) were obtained from UCSC XENA database (https://xenabrowser.net/datapages/). GTF files were obtained from Ensembl database (http://asia.ensembl.org) and were used to separate lncRNAs expression profiles from the RNAseq. We downloaded immune-related genes from ImmPort database (http://www.immport.org) and identified immune-related lncRNAs (irlncRNAs) by correlation analysis (p < 0.001, r > 0.4). Differently expressed irlncRNAs (DEirlncRNAs) were identified by intersecting irlncRNAs and differently expressed lncRNAs obtained from GEPIA2 database (http://gepia2.cancer-pku.cn/#index) in TCGA-PAAD cohort (|logFC| > 1 and FDR < 0.05).

### Making pairs of DEirlncrna

The method was previous reported [8]. In detail, we construct a X to replace paired lncRNA A and lncRNA B. The X was defined as 1 when the expression value of lncRNA A is higher than that of lncRNA B, otherwise X is defined as 0. Thus, we could get a 0-or-1 matrix. The vertical axis of the matrix represents each sample, and the horizontal axis represents each DEirlncRNA pair, with values of 0 or 1.

### Construction of the overall survival prognostic risk model

Univariate regression analysis followed by lasso regression were used to screen prognostic DEirlncRNA pairs. The lasso regression analysis was conducted with 10-fold cross validation, 1000-times repeated (p < 0.05), and the random stimulation was set up for 1,000 times in each cycle. The DEirlncRNA pairs were selected to construct prognostic risk model when the frequency of each DEirlncRNA pair in the 1000-times cycles more than 100 times. We then used AUC curve to seek the best cutoff value to group the PAAD patients into high- and low- risk groups. The AUC value of each model was also calculated and was drawn as a curve. If the curve reached the highest point, indicating the maximum AUC value, the calculation procedure was terminated while the model was taken as the optimal candidate. The 1-, 3-, and 5-year ROC curves of the model were plotted. Univariate and multivariate regression analysis were used to examine the independent prognostic efficiency of the prognostic risk model.

### Immune cell infiltration analysis

Seven tools were used to investigate the immune cell infiltration score, including XCELL, TIMER, QUANTISEQ, MCPCOUNTER, EPIC, CIBERSORT-ABS, and CIBERSORT. The immune cell infiltration data were downloaded from TIMER2 database (http://timer.comp-genomics.org/#tab-5817-3). The differences in immune infiltrating cell content explored by these methods between high- and low-risk groups of the constructed model were analyzed by Wilcoxon signed-rank test; the results are shown in a box chart. Spearman correlation analysis was performed to analyze the relationship between the risk score values and the immune infiltrated cells. The correlation coefficients of the results were shown in a lollipop diagram. The significance threshold was set as p < 0.05. The procedure was performed using R ggplot2 packages. To study the relationship between the model and the expression level of genes related to immune cell infiltration score, we performed ggstatsplot package and violin plot visualization.

### Ic50 of anti-cancer drugs

To evaluate the model in the clinic for pancreatic adenocarcinoma treatment, we calculated the IC50 of common administrating chemotherapeutic drugs in the TCGA-PAAD cohort. The difference in the half-maximal inhibitory concentration (IC50) between the high and low risk groups was compared by Wilcoxon signed-rank test and the results are shown as box drawings obtained using with pRRophetic and ggplot2 of R. All methods were performed in accordance with the relevant guidelines and regulations.

## Results

### Identification of DEirlncrnas

**Fig. 1:**
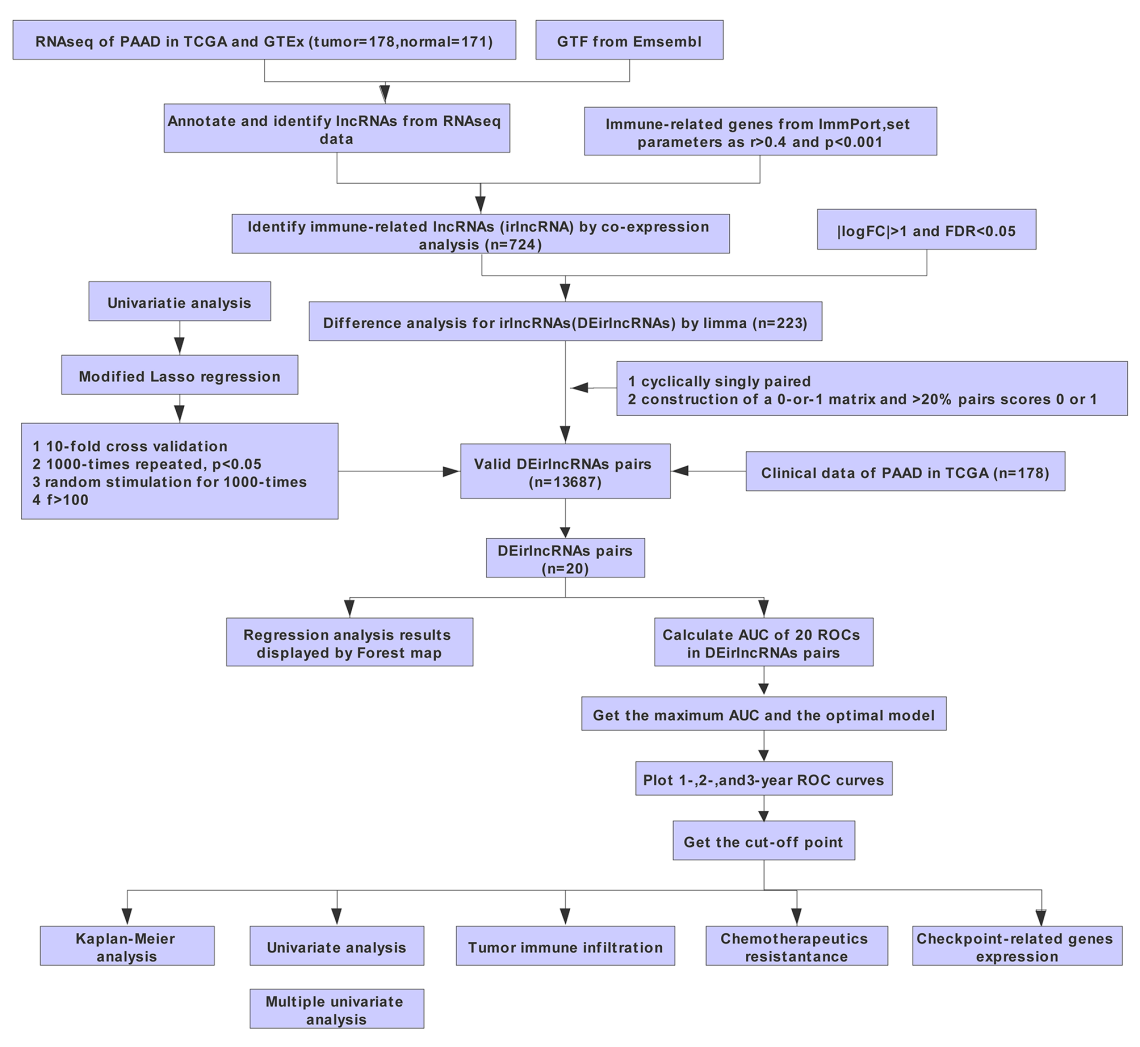
Work flow of our study

**Fig. 2:**
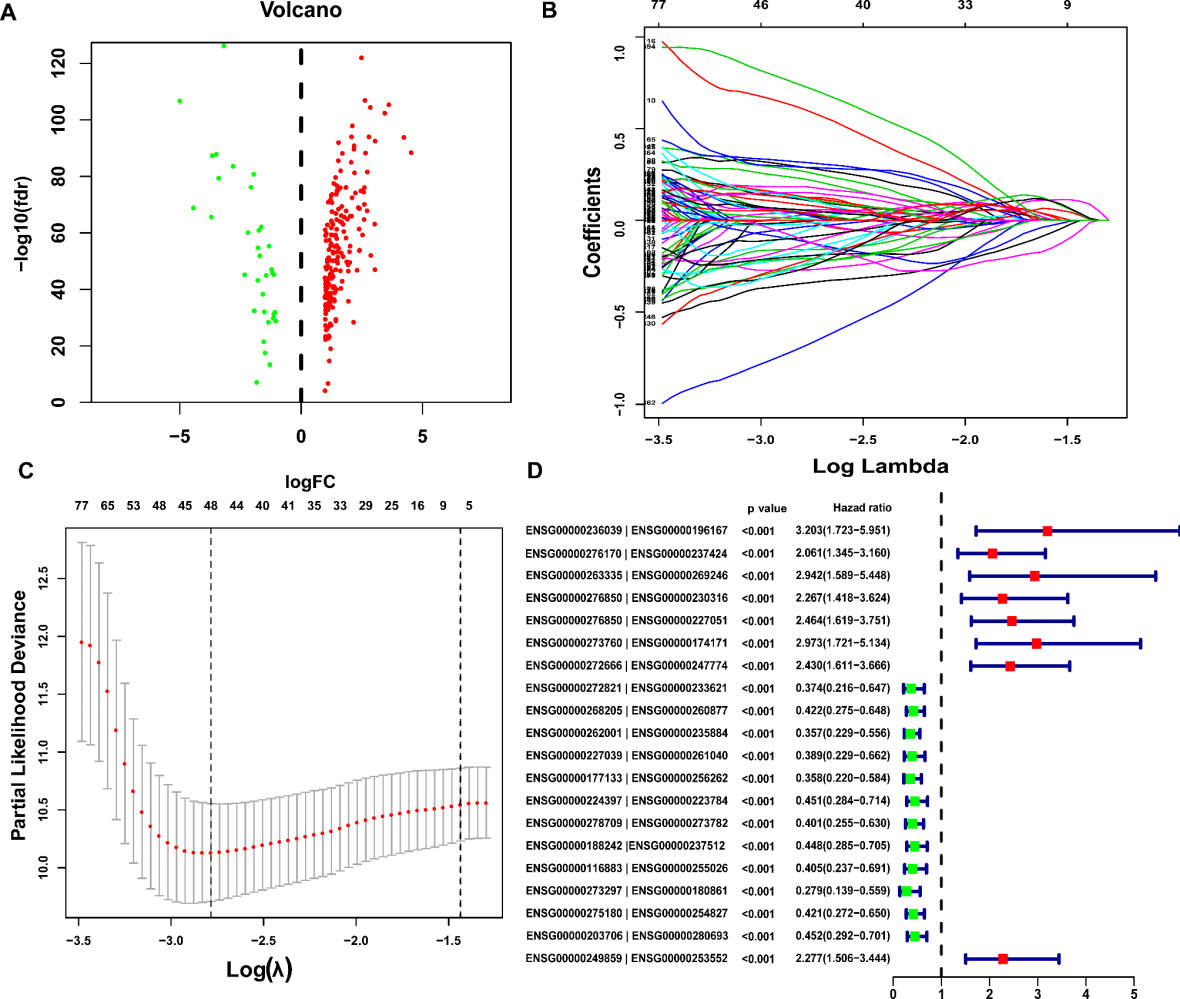
Construction of prognostic risk model. (**A**) The volcano plot of differential expressed lncRNAs. (**B**) The Distribution of the lasso coefficients for 20 DEirlncRNA pairs. (**C**) The partial likelihood deviation of the LASSO coefficient distribution. (**D**) Forest plot shows the univariate regression analysis of 20 DEirlncRNA pairs

The work flow of our research is displayed in Fig. 1. By correlation analysis between lncRNAs and immune-related genes, we screened 724 irlncRNAs with p < 0.01 and r > 0.4. We further analyzed the differential expressed lncRNAs by GEPIA2 (Fig. 2A). A total of 223 irlncRNAs were differentially expressed between pancreatic adenocarcinoma and normal pancreatic tissues (|logFC| > 1, FDR < 0.05), named DEirlncRNAs.

### Construction of prognostic risk model

**Fig. 3:**
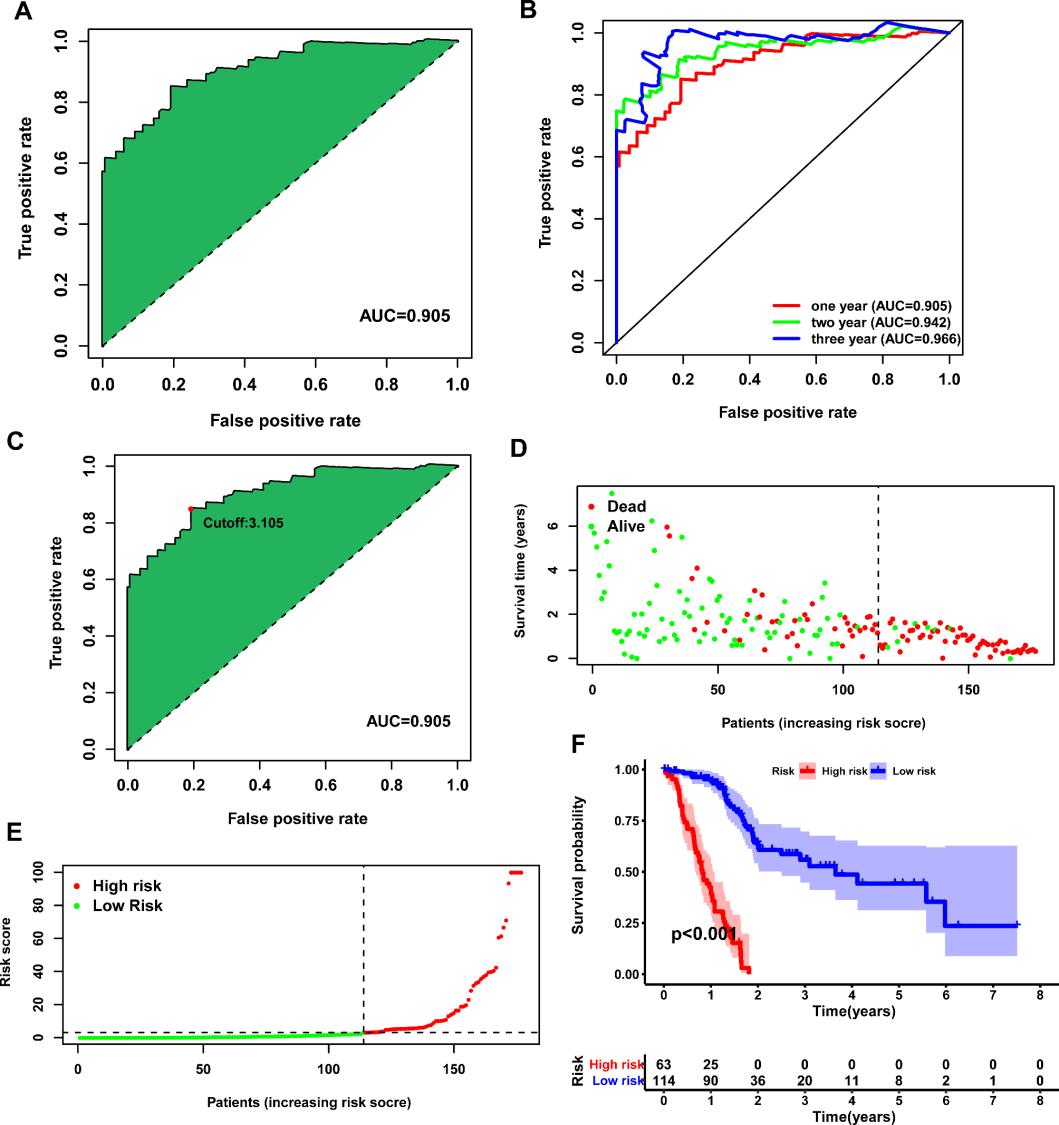
The effectiveness of prognostic risk model. (**A**) The ROC of the prognostic risk model. (**B**) The 1-, 2-, and 3-year ROC of the prognostic risk model. (**C**) The ROC of the prognostic risk model. The best cut-off point was displayed. (**D**-**E**) The distribution of the survival status (**D**) and risk scores (**E**). (**F**) Kaplan-Meier analysis of PAAD patients in high- and low-risk groups

We further constructed a 0-or-1 matrix by pairing 223 DEirlncRNAs. A total of 13,687 DEirlncRNAs pairs were identified. After the univariate and lasso regression analysis, 20 DEirlncRNAs pairs were finally screened to construct the prognostic risk model (Fig. 2B-D). According to the results of lasso and multivariate regression analysis, we calculated the risk scores of each patient in TCGA-PAAD cohort (Table 1). According to the results of lasso regression analysis, we calculated the risk scores of each patient in TCGA-PAAD cohort. The AUC for ROC curve was 0.905 for 1-year risk model prediction, 0.942 for 2-year prediction, and 0.966 for 3-year prediction (Fig. 3A-B). We set the best cutoff value 3.105 to group patients of TCGA-PAAD cohort into high- and low- risk groups and draw the survival outcome and risk score distribution of each patient (Fig. 3C-E). Kaplan-Meier analysis was used to show that PAAD patients in high-risk group have significant worse survival than patients in low-risk group (p < 0.001) (Fig. 3F).

**Table 1:** The risk scores of each patient in TCGA-PAAD cohort

id	coef	HR	HR.95 L	HR.95 H	P value
AC019117.2|COLCA1	1.00018	2.71876	1.21165	6.10053	0.01529
AC124789.1|FOXD2-AS1	0.51762	1.67803	0.96531	2.91697	0.06653
AF001548.5|CTC-246B18.10	0.48574	1.62538	0.82916	3.18618	0.15723
CH17-360D5.2|FEZF1-AS1	0.59826	1.81895	0.96362	3.43347	0.06494
CH17-360D5.2|C14orf132	0.44767	1.56467	0.92152	2.65668	0.09744
CH17-360D5.3|RP11-23P13.6	1.05298	2.86618	1.47313	5.57655	0.00193
CTA-384D8.35|PCED1B-AS1	0.81869	2.26752	1.38210	3.72016	0.00119
CTA-384D8.36|LINC01137	(0.47104)	0.62435	0.33410	1.16676	0.13981
CTC-444N24.11|RP11-211G23.2	(0.48080)	0.61829	0.35745	1.06947	0.08548
DLGAP1-AS2|LINC00941	(0.83825)	0.43247	0.24829	0.75328	0.00307
ITGB2-AS1|WFDC21P	(0.55109)	0.57632	0.31604	1.05096	0.07221
LINC00982|USP30-AS1	(0.81771)	0.44144	0.24728	0.78806	0.00568
LINC01272|RP11-554I8.2	(0.59590)	0.55106	0.32958	0.92139	0.02308
NKILA|UCA1	(0.49934)	0.60693	0.36993	0.99577	0.04807
PP7080|UNC5B-AS1	(0.56679)	0.56734	0.30669	1.04954	0.07093
RP11-268J15.5|RP11-326C3.2	(1.65764)	0.19059	0.09902	0.36684	0.00000
RP11-38M8.1|LINC01559	(0.86880)	0.41945	0.16285	1.08042	0.07190
RP11-631N16.4|SLC22A18AS	(0.56220)	0.56995	0.32388	1.00299	0.05122
SERTAD4-AS1|SH3PXD2A-AS1	(0.70243)	0.49538	0.29022	0.84557	0.01003
PVT1|HOXA-AS2	1.62170	5.06171	2.90274	8.82645	0.00000

### Association between clinical characteristics and risk scores

**Fig. 4:**
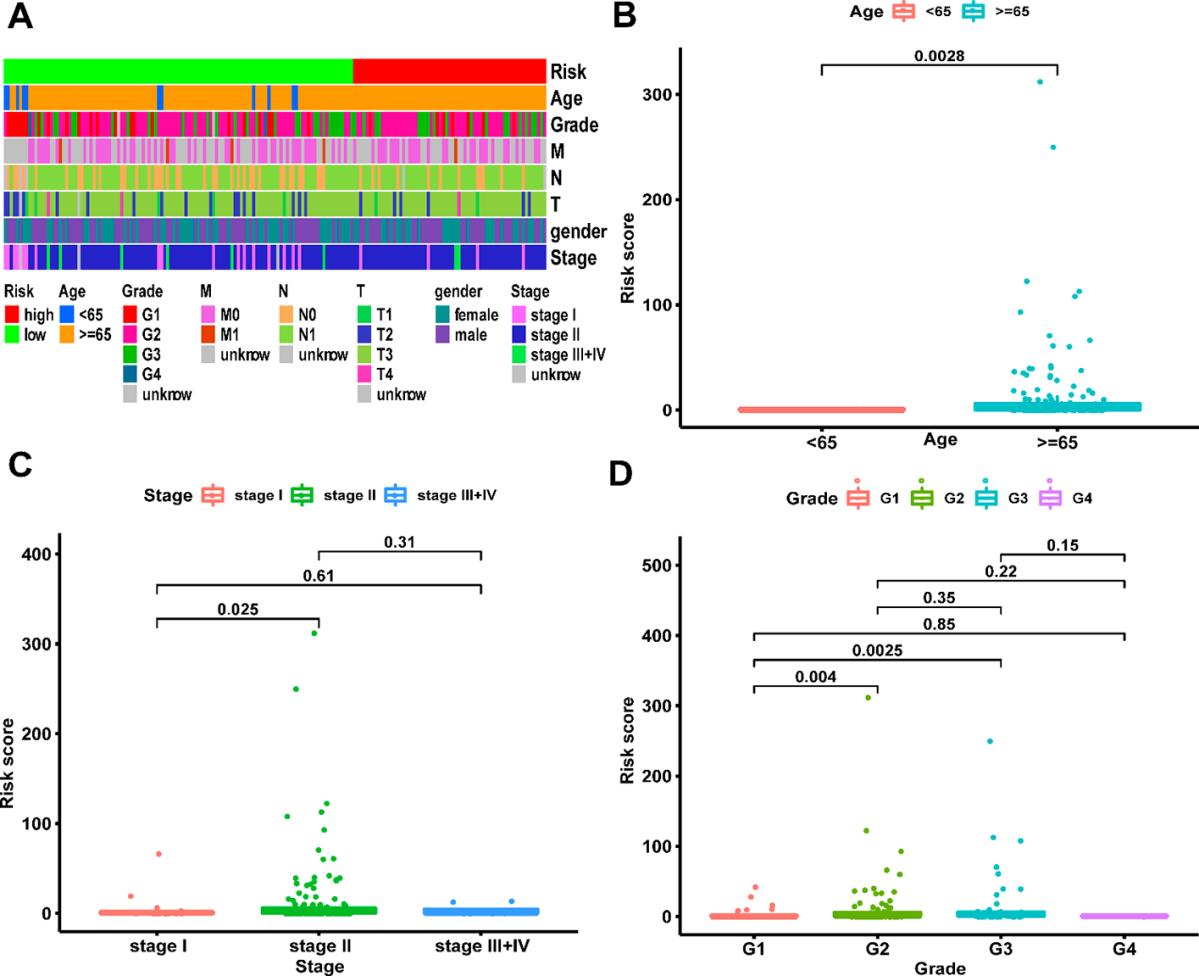
Clinical characteristics of prognostic risk model. The strip chart (**A**) showed the (**B**) ages, (**C**) tumor stages, (**D**) tumor grades, risk scores and genders of patients from TCGA-PAAD cohort. **p < 0.01

**Fig. 5:**
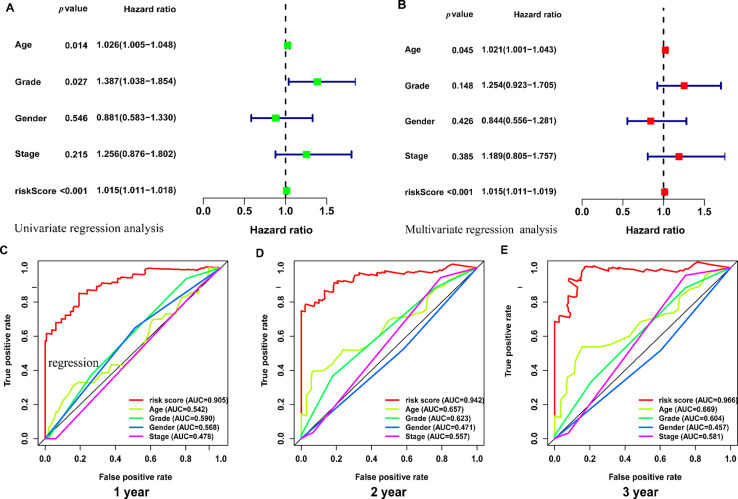
The independent prognostic analysis of prognostic risk model. (**A**-**B**) The univariate (**A**) and multivariate (**B**) regression analysis of prognostic risk model and clinical characteristics. (**C**-**E**) The 1-, 2-, and 3-year ROC of the prognostic risk model and clinical characteristics

We further assessed the difference of risk scores in different clinical characteristics. The strip chart (Fig. 4A) showed the overall association between clinical characteristics and risk scores. In detail, patients in older ages have high risk scores (Fig. 4B). In addition, the risk scores were higher in stage II patients than that in stage I patients (Fig. 4C). For tumor grades of PAAD patients, the risk scores were higher in grade 3 patients than that in grade 1 and grade 2 patients (Fig. 4D). We further performed univariate and multivariate regression analysis and proved that the risk score (p < 0.001) and age (p = 0.045) were independent prognostic factors of PAAD patients (Fig. 5A-B). The ROC curve proved that the risk score performed better than other clinical characteristics in predicting 1-, 2-, and 3-year survival of PAAD patients (Fig. 5C-E).

### Tumor immune microenvironment (TIME) analysis

**Fig. 6:**
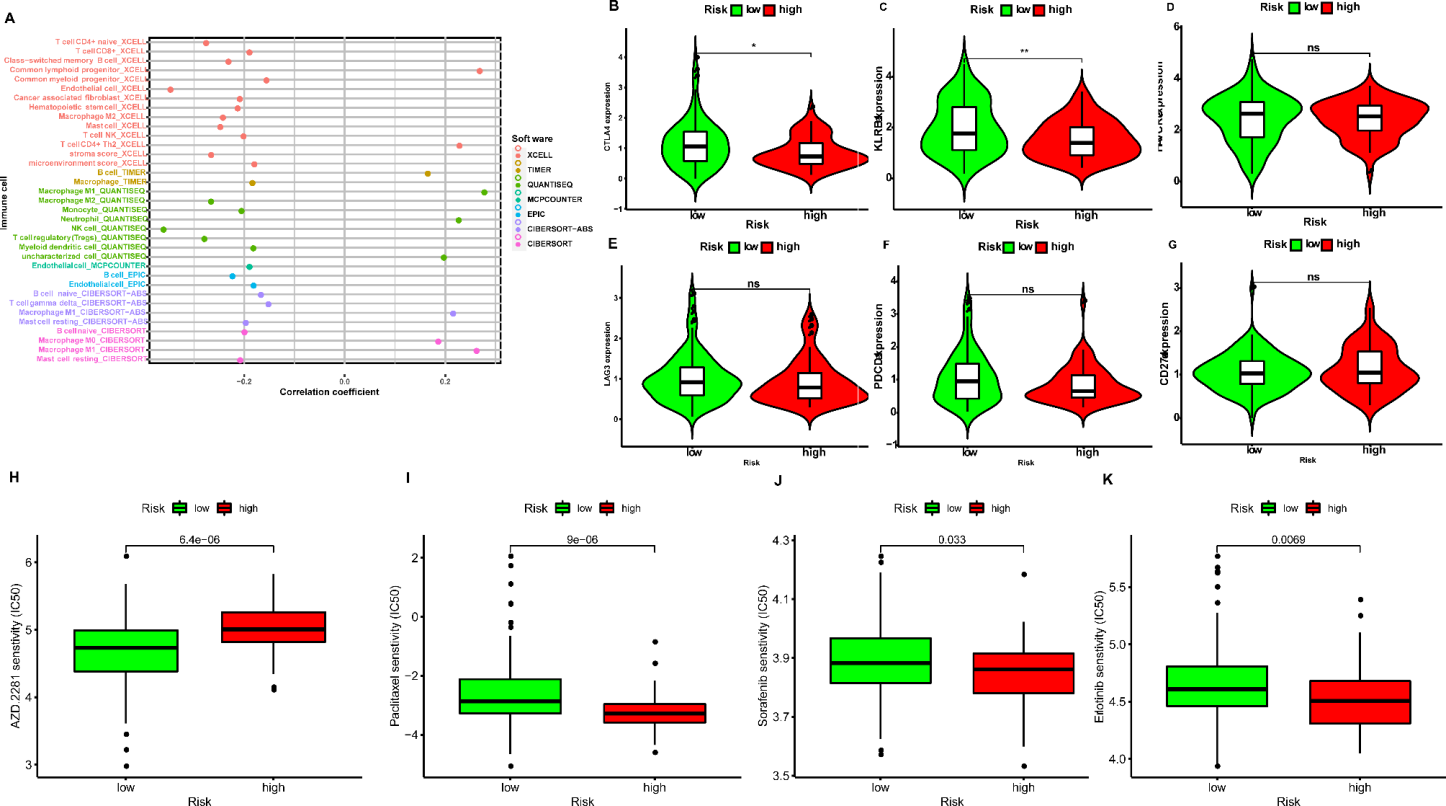
Analysis of the correlation between prognostic risk model and immune cell infiltration. (**A**) The correlation between prognostic risk model and immune cell infiltration. (**B**-**G**) Indicated gene expression in high- and low-risk groups. (**H**-**K**) The IC50 values of indicated anti-cancer drugs in high- and low-risk groups. *p < 0.05, **p < 0.01, ns = non-significant

**Fig. 7:**
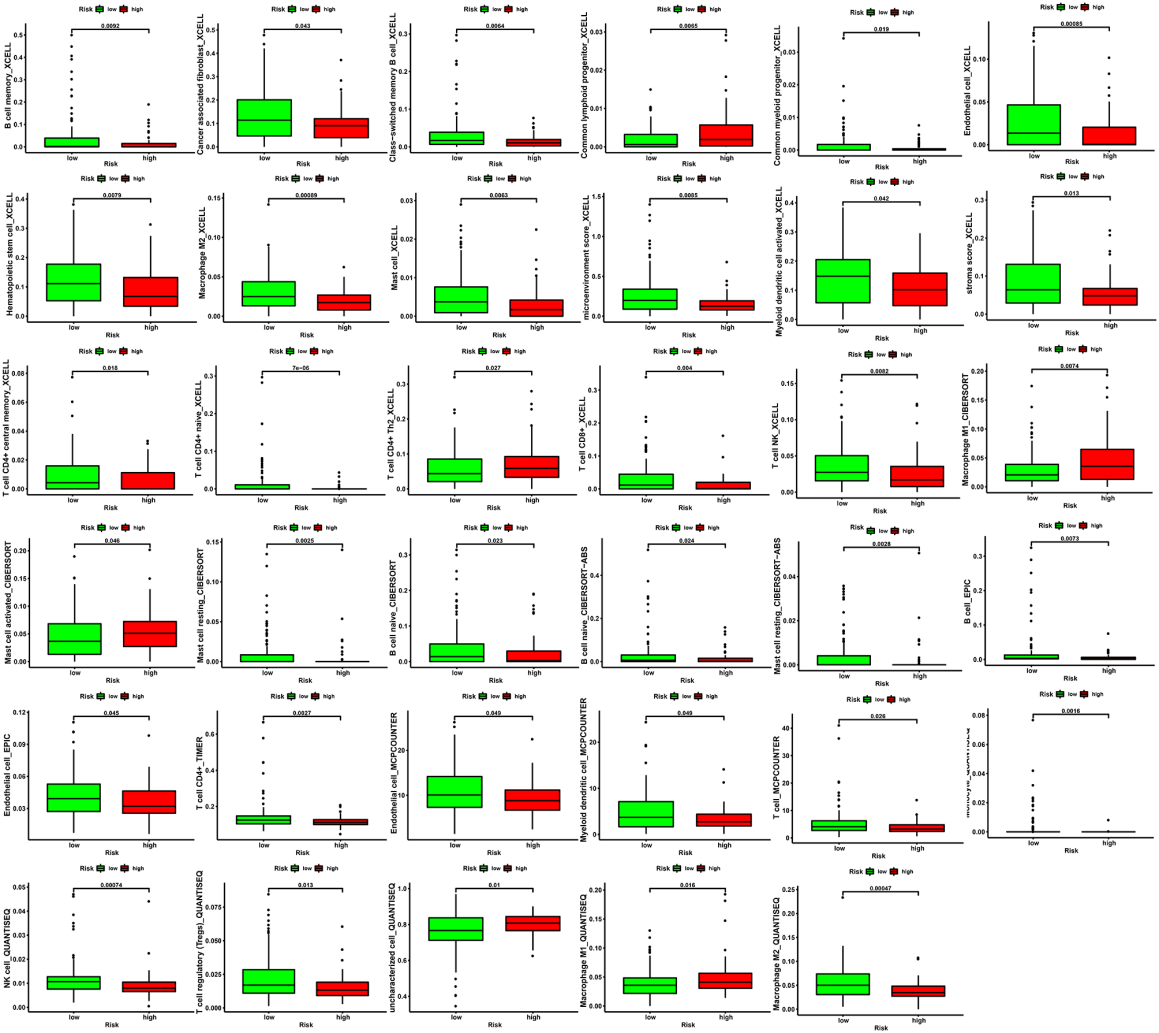
The differences of immune cell infiltration in high- and low-risk groups

We consequently explored the association between TIME and risk scores. We found that the risk scores of PAAD patients were negatively correlated with CD8^+^ T cells and NK cells (Fig. 6A), indicating that the immune function was suppressed in high-risk groups. We also evaluated the differences of immune cell infiltration in high- and low-risk groups and got the same results (Fig. 7). The infiltration of CD8^+^ T cells and NK cells were lower in high-risk group. In recent years, immune checkpoint inhibitors (ICIs) have been widely used in the treatment of solid tumors. However, the use of ICIs in pancreatic adenocarcinoma is rarely successful. Thus, we assessed the immune checkpoint genes expression in high- and low-risk groups. We found that CTLA-4 and CD161 (KLRB1) were over-expressed in low-risk group (Fig. 6B-G), indicating that PAAD patients in low-risk group may be sensitive to ICIs.

### Correlation between the chemoresistance and risk scores

We further evaluated the correlation between risk scores and common chemotherapeutics in TCGA-PAAD cohort. We searched the commonly anti-tumor drugs in pancreatic cancer and analyzed the differences of their IC50 values between high- and low-risk groups. Results revealed that the IC50 value of AZD.2281 (Olaparib) was higher in high-risk group, indicating that PAAD patients in high-risk group may be resistant to AZD.2281 treatment (Fig. 6H). In addition, the IC50 values of Paclitaxel, Sorafenib, and Erlotinib were lower in high-risk groups (Fig. 6I-K). We further identified 34 anti-cancer drugs with their IC50 values higher in high-risk group and 34 anti-cancer drugs with their IC50 values lower in high-risk group (Table 2).

**Table 2:** The association between anti-cancer drugs and risk scores of PAAD patients

Drugs with elevated IC50 values in high-risk group	Drugs with reduced IC50 values in high-risk group
PD.173,074 NVP.BEZ235 MK.2206 Mitomycin.C Methotrexate Vorinostat Lenalidomide PD.0332991 (Palbociclib) CEP.701 (Lestaurtinib) AZD8055 ATRA Temsirolimus AP.24,534 (Ponatinib) ABT.888 (Veliparib) SB.216,763 TW.37 Axitinib Metformin GDC.0449 EHT.1864 CCT007093 Camptothecin AMG.706 (Motesanib) VX.702 SB590885 QS11 ZM.447,439 BIRB.0796 Salubrinal Nutlin.3a NU.7441 KU.55,933 Elesclomol Nilotinib	GNF.2 Lapatinib Dasatinib AZD.0530 (Saracatinib) RDEA119 PD.0325901 AICAR AKT.inhibitor.VIII AUY922 (Luminespib) CI.1040 Bryostatin.1 Bortezomib BIBW2992 (Afatinib) A.443,654 Thapsigargin VX.680 BMS.536,924 CMK BMS.509,744 PHA.665,752 Bicalutamide PF.562,271 A.770,041 Epothilone.B GW843682X WZ.1.84 X17.AAG Z.LLNle.CHO CGP.082996 FTI.277 NSC.87,877 JW.7.52.1 S.Trityl.L.cysteine BI.2536

## Discussion

There is no denying that lncRNAs, mRNAs and miRNAs are all extensive, playing crucial roles in cancer. There were ample evidences supporting crucial roles of mRNAs or miRNAs in predicting overall survival in multiple cancer types. Undoubtedly, many prognostic risk models were also based on lncRNAs. For example, Luo et al. found that LINC01094 plays critical roles in proliferation and metastasis of PC, high expression of LINC01094 indicated worse survival of pancreatic cancer patients [16]. The study presented by Lin et al. revealed that lncRNA FLVCR1-AS1 downregulation was associated with a worse prognosis in patients with pancreatic cancer [17]. However, immune-related lncRNAs were relatively seldom discussed in predicting overall survival of cancer patients. Recently, a body of work has focused on construction of prognostic risk models to predicted the survival of tumor patients, thus to adjust treatment methods [18–20]. The role of immune infiltration is increasingly recognized to be important in cancer development, progression, and response to therapies such as chemotherapy. Multiple studies have confirmed that tumor-infiltrating immune cells play crucial roles in response to cytotoxic chemotherapy [21–23]. The tumor immune microenvironment was a vital factor in survival of tumor patients [24, 25]. Immunotherapy, especially ICIs treatment, have been widely used in the treatment of solid tumors [26]. The immune-related genes were widely used to construct prognostic risk models. For example, Su et al. established an immune-related prognostic risk model based on protein-coding genes, which could predict the prognosis of patients with ovarian cancer [27]. Non-coding genes like lncRNAs were also suitable for construction of prognostic risk models [28–30]. Luo et al. screened 4 immune-related lncRNAs and constructed a prognostic risk model in cervical cancer [31]. Han et al. identified a total of 32 differential expressed transcripts and based on that, a predictive model with five significant transcripts was established, which was suggested as a highly recommended tool for the prediction of biopsy-proven acute rejection after kidney transplantation [32].

Most of these models were based on gene expression level, no matter of protein-coding genes or non-coding genes. However, the same gene expression values may be different in different gene sets, data forms and patients, inducing the scores of prognostic models unstable. In this study, we construct a reasonable model with two-lncRNA pairs, independent on the exact expression values.

In this study, we first identified irlncRNAs by correlation analysis with immune-related genes. By crossing with differential expression lncRNAs, we screened 223 DEirlncRNAs. Second, we constructed a 0-or-1 matrix based on a published DEirlncRNAs pairing method [31]. Next, we conducted univariate and lasso regression analysis to determine prognostic DEirlncRNAs pairs and established a prognostic risk model. We further analyzed the association between risk scores and clinical characteristics of PAAD patients. We found that our prognostic risk model, as an independent prognostic factor in PAAD patients, could effectively distinguish high-stage patients from low-stage patients and high-grade patients from low-grade patients. Moreover, the AUC value for ROC curve of the prognostic risk model was 0.905 for 1-year prediction, 0.942 for 2-year prediction, and 0.966 for 3-year prediction.

Researchers have reported that patients with high CD8^+^ T cells infiltration were more sensitive to ICIs treatment [33]. The increased the content of cytotoxic cells, NK CD56 cells, NK cells and CD8 + T cells in the tumor immune microenvironment may be one reason for the tumor-inhibiting effect [34]. Previous studies have found that higher levels of tumor-infiltrating CD4(+) T, CD8(+) T, were significantly associated with longer survival [35]. Poor CD8 T-cell infiltration, low neoantigen load and a highly immunosuppressive tumor microenvironment contribute to lack of response to ICIs treatment [36]. We found that the risk scores were negatively correlated with CD8^+^ T cells and NK cells, indicating that patients with high-risk scores may be not suitable for ICIs treatment and they have a worse prognosis.

CD161 is a marker of natural killer (NK) cell. CD8 + CD161 + CAR-transduced T cells mediated enhanced in vivo antitumor efficacy in xenograft models of HER2 + pancreatic ductal adenocarcinoma [37]. Immune checkpoint inhibitors, targeting cytotoxic T-lymphocyte-associated protein 4 (CTLA-4) and the programmed cell death protein-1 (PD-1)/programmed cell death ligand-1 (PD-L1) pathways have shown remarkable potential in several types of cancer [38].The expression of CTLA-4 and CD161 (KLRB1) were lower in high-risk groups, which also indicated that patients with high-risk scores may be not suitable for ICIs treatment.

To seek the suitable treatment for high-risk patients, we analyzed many anti-cancer drugs and found that PAAD patients in high-risk groups may be suitable to Paclitaxel, Sorafenib, and Erlotinib, which were widely used in PAAD patients [33]. Zhang et al., found that mutation in any DNA damage response (DDR) pathway results in a poor prognosis for prostate cancer patients [39]. The Pancreas Cancer Olaparib Ongoing (POLO) trial, demonstrated that Olaparib maintenance therapy prolongs progression-free survival compared to placebo in patients with pancreatic ductal adenocarcinoma and germline BRCA1/2 mutations after platinum-based first-line chemotherapy [40]. This has generated considerable optimism regarding substantially improved outcomes for this patient subgroup. In this study, the IC50 value of AZD.2281 (Olaparib) was higher in high-risk group, indicating Pthat PAAD patients in high-risk group may be resistant to AZD.2281 treatment.

The predictive models in this study have good predictive results, but these are based on analytical predictions. How to confirm these results in clinical data is an important issue. Endoscopic ultrasound with fine-needle aspiration (EUS-FNA) has evolved into an indispensable diagnostic modality for solid pancreatic and extra-pancreatic lesions with a sensitivity of 85% and specificity of 98% [41]. The advent of EUS fine-needle biopsy (EUS-FNB) needles was primarily premised on putative benefits over FNA such as higher diagnostic accuracy, procurement of specimens with preserved histological architecture thereby enabling immunohistochemistry or special stains pivotal for certain diagnoses [42]. A systematic literature review confirmed that FNB needles particularly with 22G size, showed the highest performance for tissue sampling of pancreatic masses [43]. In clinical practice, only a small number of patients can undergo radical surgery, and most patients have unresectable tumors at the time of initial diagnosis. In clinical practice, only a minority of patients are amenable to curative surgery, as most patients present with unresectable tumors at initial diagnosis. After securing pathological confirmation via EUS-FNB and other modalities, standardized nonsurgical therapies such as chemotherapy are commonly opted for. Our subsequent research agenda is to validate the predictive model of this study in both surgical and non-surgical cohorts through retrospective analyses.

## Conclusions

Overall, our study constructed a novel prognostic risk model based on pairing irlncRNAs, exhibited a promising prediction value in patients with pancreatic adenocarcinoma. Our prognostic risk model may help distinguish PAAD patients suitable for medical treatments.

## Data Availability

The datasets used and analyzed during the current study available from the corresponding author on reasonable request.
